# Bridging the Gap in Racial/Ethnic Disparities: Perspectives from the 2025 American Heart Association/American College of Cardiology High Blood Pressure Guideline

**DOI:** 10.1007/s11906-026-01367-6

**Published:** 2026-02-20

**Authors:** Tina K. Reddy, Bhavya Ancha, Jelani K. Grant, Keith C. Ferdinand

**Affiliations:** 1 Department of Medicine, Johns Hopkins University School of Medicine, Baltimore, MD USA; 2 Division of Cardiology, Department of Medicine, Johns Hopkins Ciccarone Center for the Prevention of Cardiovascular Disease, Johns Hopkins School of Medicine, Baltimore, MD USA; 3 Section of Cardiology, John W. Deming Department of Medicine, Tulane University School of Medicine, 1430 Tulane Ave, New Orleans, LA 70112 USA

**Keywords:** Blood pressure, Hypertension, Social determinants of health, Racial/ethnic disparities, AHA/ACC high blood pressure guideline

## Abstract

**Purpose of Review:**

High blood pressure is a leading cause of cardiovascular disease (CVD) morbidity and mortality worldwide, disproportionately affecting racial/ethnic populations in the United States (US). This review summarizes racial/ethnic inequities in CVD risk, hypertension (HTN), and the social determinants or drivers of health (SDOH), highlighting the 2025 American Heart Association/American College of Cardiology (AHA/ACC) High Blood Pressure Guideline.

**Recent Findings:**

Certain US racial/ethnic populations experience disproportionate HTN burden, with Black adults demonstrating higher rates of resistant, nocturnal and masked HTN, hypertensive disorders of pregnancy, and dementia and cognitive decline. The 2025 AHA/ACC High Blood Pressure Guideline emphasizes the use of a novel race-neutral CVD risk calculator to guide initiation of pharmacotherapy, utilizing patient counseling and shared decision-making, alongside team-based, community-engaged HTN management strategies.

**Summary:**

The 2025 AHA/ACC High Blood Pressure Guideline advances health equity, integrating the SDOH into improved CVD risk assessment and management recommendations. Effective mitigation of disparities to improve blood pressure and CVD outcomes demands optimizing healthcare access, addressing systemic discrimination, incorporating social context into shared clinical-decision making, and deploying culturally-tailored, community-based interventions.

## Introduction

High blood pressure (HBP) is a major global health crisis, with prevalence doubling from 650 million in 1990 to 1.3 billion in 2019 [1]. Furthermore, it is the most prevalent and modifiable risk factor for cardiovascular disease (CVD), including coronary artery disease (CAD), heart failure (HF), atrial fibrillation, chronic kidney disease (CKD), stroke, dementia and all-cause mortality [2]. Elevated systolic blood pressure (SBP) accounts for an estimated 10.8 million deaths and over 235 million disability-adjusted life years each year—more than tobacco use or hyperglycemia [1]. Nevertheless, HBP is largely preventable and manageable through cost-effective interventions, making its control essential to achieving global health targets. In the United States (US), uncontrolled hypertension (HTN) imposes a substantial economic burden, with estimated annual healthcare costs ranging from $131 to $198 billion [3]. Despite the availability of effective therapies, HTN control rates in the US have declined over the past decade, with significantly lower control observed among racial/ethnic groups.

Furthermore, in the US, non-Hispanic Black (NHB) adults experience earlier onset and disproportionately higher rates of HTN-related complications and mortality compared to non-Hispanic White (NHW) individuals [4]. In addition, HTN disparities are also evident among Asian, Hispanic, and American Indian/Alaska Native (AI/AN) populations. Among those receiving antihypertensive therapy, Asian adults are less likely to receive intensive treatment and have lower rates of blood pressure (BP) control, with Hispanic adults demonstrating similar patterns [5]. American Indian and Alaska Native populations experience higher prevalence of HTN and lower rates of BP control, with substantial regional variation, contributing to excess CVD burden, resulting in earlier onset and higher mortality from CVD [6, 7]. Notably, among treated individuals, Asian adults have the greatest burden of uncontrolled BP, exceeding rates observed among Black, Hispanic, and White adults [7]. These disparities translate into adverse outcomes, as analyses of large Asian American subgroups demonstrate a higher proportion of deaths attributable to hypertensive disease across all subgroups when compared with NHW women [8]. Nevertheless, beyond self-identified race or ethnicity, which are social constructs, disparities in optimal HTN control are primarily driven by social determinants or drivers of health (SDOH), including lower income, lower educational attainment, neighborhood disadvantage, limited access to care, low social support, and discrimination, which augment the risk of uncontrolled HTN across diverse racial/ethnic populations [9].

The 2025 American Heart Association/American College of Cardiology (AHA/ACC) Guideline for the Prevention, Detection, Evaluation and Management of High Blood Pressure in Adults: A Report of the AHA/ACC Joint Committee on Clinical Practice Guidelines (2025 AHA/ACC HBP Guideline) introduces several updates relevant to addressing these inequities. Notably, it is the first HTN or cardiovascular guideline to adopt the AHA Predicting Risk of Cardiovascular Disease EVENTs (PREVENT) calculator as a tool for risk assessment and treatment initiation. The PREVENT calculator excludes race, acknowledging its limitations as a biological construct, and incorporates ZIP code as a contextual measure of the SDOH. Although imperfect, this approach reflects a shift toward risk prediction grounded in contemporary, diverse datasets and environmental factors that more closely align with observed disparities.

This review examines racial/ethnic disparities in HTN prevention and management, with particular attention to key updates in the 2025 AHA/ACC HBP guideline. Specifically, this review will (1) summarize the disparate burden of HTN and recent trends in HBP control; (2) examine persistent racial/ethnic disparities in HTN subtype, including in nocturnal HTN, masked HTN, resistant HTN, hypertensive disorders of pregnancy (HDPs), and dementia and cognitive decline; (3) evaluate how key guideline updates, including integration of the PREVENT tool, address inequities in care and outcomes; and (4) discuss implications for improving equitable HTN prevention and management. Although most available data on HTN disparities are derived from US populations, this review also briefly comments on recent international guidelines that may offer additional insights.

## Distinct Patterns and Drivers of Racial/Ethnic Disparities by Hypertension Sub-Type

Racial/ethnic disparities in HTN are well documented in the US. Hypertension disproportionately affects NHB adults (56.8% of men; 56.7% of women), non-Hispanic Asian adults (49.8% of men; 39.1% of women), and Hispanic adults (50.4% of men; 36.3% of women), relative to NHW adults (47.0% of men; 39.0% of women) [2]. Overall, Black adults also experience high rates of HTN-related complications, including end-stage kidney disease (4 times higher than White men), stroke, left ventricular hypertrophy, HF, and HTN-related mortality compared to their White counterparts (4–5 fold higher) [10]. Notably, while Black adults demonstrate similar or higher rates of HTN awareness and treatment compared to White adults, control rates remain lower. This discrepancy is likely due to underrecognition, undertreatment, and adverse environmental conditions. Globally, Black populations, including those in the US, Africa, the Caribbean, and Europe, may develop HTN and related end-organ damage at younger ages, with a higher prevalence of nocturnal, masked, and resistant HTN.

In the US, nocturnal hypertension (NH), defined as elevated BP during sleep, is primarily identified using ambulatory blood pressure monitoring and affects more than 20% of White adults and 40% of Black adults [11]. It is increasingly recognized as a strong predictor of adverse cardiovascular (CV) events, perhaps even more so than daytime BP [12]. In a cross-sectional study (*N* = 1,585, 7.9% NHB), 41.3% of adults exhibited co-occurrent NH and nondipping BP. These abnormalities were more prevalent among men, NHB patients, and individuals with CKD or HTN, suggesting that these factors contribute to elevated CV risk beyond that captured by standard BP assessments.

Masked HTN, defined as elevated out-of-office but normal office BP (< 140/90 mm Hg), affects approximately 15–30% of people, representing an estimated 17 million US adults [11]. When NH is included, prevalence exceeds 50% among Black individuals. Individuals with office systolic/diastolic BP within 20/10 mm Hg of the HTN threshold are at particularly high risk for masked HTN [11].

Resistant hypertension (RH) is defined as BP that remains above target despite treatment with 3 antihypertensive medications with complementary mechanisms of action, including a diuretic (at maximally tolerated doses) or BP at goal but requiring ≥ 4 medication. Moreover, RH is associated with increased CV risk and disproportionately affects NHB individuals [2, 13]. Black race consistently identified as one of the strongest predictors of RH across large cohort studies [13]. The main contributor to RH is often inadequate treatment. Among Black adults with apparent RH, there may be suboptimal lifestyle measures and underutilized effective pharmacotherapy, including chlorthalidone, indapamide, or mineralocorticoid receptor antagonists [13]. This highlights a significant gap in implementing evidence-based lifestyle and pharmacologic interventions in this population.

Hypertensive disorders of pregnancy (HDPs) are another major contributor to maternal and neonatal mortality and morbidity, affecting 15.9% of US deliveries, having culpability for 1 in 3 delivery-associated hospitalization deaths and causing 6.8% of pregnancy-related deaths overall [14, 15]. Prevalence is highest among NHB, AI/AN women, women aged 35 years and older, and individuals with obesity [2]. Patients with HDPs have higher risk of chronic HTN, CAD, HF, and stroke [16, 17]. This underlines HDPs as an early marker of later adverse CVD outcomes. The American College of Obstetricians and Gynecologists classifies Black race as a moderate risk factor for preeclampsia, recognizing that this association reflects the health consequences of structural racism and systemic inequities rather than inherent biological differences [18]. Evidence demonstrates that initiating daily low-dose aspirin after 12 weeks of gestation significantly reduces the risk of preeclampsia among individuals at moderate to high risk. Consistent with these findings, the 2025 AHA/ACC High Blood Pressure Guideline issues a Class IB recommendation for low-dose aspirin (81 mg/day) in patients with HTN who are planning pregnancy or are pregnant, emphasizing counseling on its benefits in lowering the risk of preeclampsia and related maternal and fetal complications [2]. The observed disparities in maternal health further emphasize the need for early risk identification and intervention in high-risk populations.

## Cerebrovascular Health: Cognitive Decline and Dementia in Adults

More than 750,000 US adults experience a stroke each year, and over 75% of survivors of ischemic or hemorrhagic stroke have coexisting HTN [19]. Despite the high prevalence of HTN among stroke survivors, BP control in this group remains suboptimal, with the lowest control rates observed in Black and Hispanic adults. Racial differences in stroke incidence and outcomes are well documented: Black adults experience earlier stroke onset, higher rates of recurrent cerebrovascular events, and greater post-stroke disability compared with White adults [20–22]. Current evidence supports targeting BP levels < 130/80 mmHg for secondary stroke prevention in patients with established cerebrovascular disease [2].

Black adults remain markedly underrepresented in Alzheimer’s disease and dementia research and clinical trials. Yet, with the data available, the burden of Alzheimer’s disease and related dementias (ADRD) is highest in Black populations [23, 24]. Among Black Americans ≥ 70 years, 21.3% are living with Alzheimer’s disease, twice the rate seen in older White adults. Access to high-quality dementia care remains inequitable; only 20% of Black Americans report having no barriers to receiving quality Alzheimer’s care and just 48% feel confident they can access culturally competent providers [25]. Consistent with these disparities, analysis of a US veteran population demonstrated that age-adjusted dementia incidence rates were highest among Black and Hispanic participants, with rates similar among AI/AN, Asian, and White participants [24].

Black women, particularly those over age 65, are at even greater risk of ADRD, yet they are 35% less likely to receive a diagnosis compared to White women, often due to underdiagnosis, discrimination, and systemic inequities. In addition, common modifiable risk factors, including obesity, HTN, and type 2 diabetes (T2D), are more prevalent among Black women [26]. Underrepresentation in clinical trials, where fewer than 10% of participants are NHB, further limits generalizability of current evidence, while longstanding mistrust of medical research continues to undermine participation and equitable dementia care [26].

## The Social Determinants/Drivers of Health and Barriers to Blood Pressure Control

The SDOH, or social drivers of health, are central to disparities in HTN and CVD, particularly among NHB and Hispanic populations [27]. Racial disparities in BP control are not rooted in biological differences but largely reflect adverse SDOH and experiences of racism [28]. Higher education, insurance coverage, income, and favorable neighborhood conditions are associated with lower HTN prevalence and better BP control, whereas pharmacy closures, systemic barriers, and structural racism exacerbate disparities in HTN [29–31].

Individual-level obstacles, including poor treatment adherence, mental health challenges, and difficulties with BP self-monitoring, interact with clinical and system-level challenges such as therapeutic inertia, time constraints, implicit bias, inaccurate in-office BP measurements, limited community resources (e.g. safe exercise spaces or access to healthy food), and guideline complexity. Policy-level gaps, such as inadequate insurance coverage, further hinder BP control. Medicaid expansion, culturally tailored health interventions, and community health worker programs have shown promise in improving outcomes and reducing costs [32, 33]. The 2021 and 2025 AHA/ACC HBP guidelines and American Medical Association (AMA) recommend comprehensive strategies including anti-racism initiatives, team-based care, lifestyle modification, and promotion of medication adherence to address these intersecting challenges [28].

Area-level measures based on zip code can obscure meaningful within-area heterogeneity and may misclassify social vulnerability. Geographic units such as census tracts or block groups may provide more granular and accurate assessments of contextual deprivation. To address the growing gap between guideline recommendations and real-world implementation, structurally embedded solutions, such as electronic health record (EHR)-integrated risk calculators, streamlined clinical decision-support tools, shared decision-making frameworks, quality-linked incentives and robust team-based care models emphasized in the 2025 AHA/ACC HBP Guideline are increasingly necessary [34].

Racial discrimination and chronic psychosocial stress further elevate HTN risk among NHB adults, contributing to care avoidance, delayed diagnosis, and worse outcomes [35]. The 2025 AHA/ACC HBP guideline notes that breathing exercises and yoga may modestly reduce BP and serve as adjuncts to lifestyle or pharmacologic therapy, especially in high-risk adults, though evidence is limited and variable [2]. These interventions should complement, not replace, evidence-based pharmacotherapy as indicated.

Understanding the SDOH has evolved from early socioeconomic status-based stratification (1930s) to comprehensive indices like the Healthiest Communities Index (2010s), with efforts underway to develop next-generation, CVD specific SDOH tools integrating area-level deprivation and population risk into clinical decision-making [36]. Ultimately, achieving CV health equity will require not only better tools and data but also intentional disruption of outdated structures, removal of bias from clinical algorithms, and development of inclusive, patient-centered policies and care models [36].

## Modernizing Cardiovascular Risk: PREVENT

The AHA PREVENT equations were developed to address limitations of earlier CVD risk models, including risk overestimation and reliance on race-based variables [37]. The PREVENT calculator offers both 10- and 30- year risk estimates and integrates kidney-related variables to better capture the intersection of CV, kidney, and metabolic health, while enabling risk estimates for overall CVD and its components (e.g. atherosclerotic cardiovascular disease, HF, coronary heart disease, and stroke) [38]. The equations were derived from a contemporary dataset of 3.2 million adults (1992–2022) with broader demographic representation (56% women; mean age 53 years; with 3% Asian, 5% Hispanic, and 13% Black participants) than legacy cohorts. A subsequent validation in over 361,000 real-world patients, including one-third Asian or Hispanic patients, demonstrated robust discrimination and calibration across racial and ethnic groups, with marginally better performance in Asian and Hispanic subpopulations [37]. These findings support the generalizability of PREVENT, even among previously underrepresented populations. It also integrates clinical variables and optional measures such as hemoglobin A1c, urine albumin-creatinine ratio, and the social deprivation index (SDI). Nonetheless, its accuracy may vary by population and clinical setting, underscoring the need for local validation and individualized interpretation of risk tools [39]. The PREVENT “risk age” model was developed to enhance risk communication by translating absolute risk into a more intuitive metric, risk age: the age of an individual with optimal risk factors but equivalent predicted CVD risk [40]. This disproportionate impact of adverse SDOH, particularly in racial and ethnic minority groups, may highlight individuals with elevated lifetime risk who may not meet short-term treatment thresholds.

In alignment with the 2025 AHA/ACC HBP Guideline, PREVENT supports a risk-based strategy for initiating antihypertensive therapy at a 10-year CVD risk threshold of ≥ 7.5% and allows earlier intervention in younger adults, a critical consideration for groups such as Black adults, among whom nearly 25% develop HTN before age 30 [41]. Although PREVENT removes race, it continues to assign higher predicted risk to Black adults due to their greater burden of comorbidities and structural disadvantage, illustrating that race neutrality does not eliminate underlying inequities. Limitations remain, including incomplete capture of the SDOH and imprecision of geographic proxies such a ZIP code. For instance, in one Michigan analysis, nearly half (49%) of the population were assigned to the wrong municipality using ZIP codes alone [42]. Such misalignment can distort environmental exposure assessments and health outcomes, indicating the need for more precise geographic identifiers in public health research.

In PREVENT, the 2021 CKD-EPI (Chronic Kidney Disease Epidemiology Collaboration) race-free equation for estimating glomerular filtration rate is mandated. The updated equation improves accuracy compared with older formulas and eliminates the race-based correction factor, which previously overestimated estimated glomerular filtration rate in Black patients. Its adoption may help reduce disparities in referrals to nephrology and evaluation for renal transplantation, further supporting efforts to remove racial bias in clinical care.

Additionally, CV risk may be underestimated in certain groups, such as South Asian individuals, due to non-biological factors not captured in existing US models, highlighting the need for more representative and disaggregated data [43]. Conversely, widely used tools like the pooled cohort equation and Framingham scores tend to overestimate coronary heart disease risk in East Asian populations, where stroke is more common, prompting development of locally calibrated models such as Systematic COronary Risk Evaluation 2 in Asia (SCORE2-ASIA) [44]. However, these tools often lack external validation and do not fully incorporate risk-enhancing factors or adequately represent Asian American populations. The Multi-ethnic Observational Study in American Asian and Pacific Islander Communities (MOSAAIC) study, launched in 2023, aims to address these gaps by enrolling 10,000 Asian American and Pacific Islander participants, providing data essential for improving precision and equity [44].

## Unique Aspects of Lifestyle and Practical Approaches to Control Blood Pressure Across Racial/Ethnic Populations

The 2025 AHA/ACC HBP guideline emphasizes that primary HTN arises from a complex interplay of genetic factors, lifestyle behaviors, and chronic stress [2]. Importantly, even among individuals with elevated genetic risk, adherence to healthy lifestyle practices can mitigate development of HTN. Despite strong evidence, lifestyle modification remains a foundational yet frequently underutilized component of HTN prevention and treatment. The Dietary Approaches to Stop Hypertension (DASH) diet - endorsed by the AHA, ACC, and American Diabetes Association - prioritizes high intake of fruits, vegetables, low-fat dairy products, and whole grains, while limiting saturated fat, sodium, and added sugars. Clinical trials have demonstrated that adherence to the DASH diet can reduce SBP by up to 11 mmHg, with additional metabolic and vascular health benefits [2]. This diet is particularly effective in Black adults and individuals with elevated BP, due to its nutrient rich profile in potassium, calcium, magnesium, and fiber. However, dietary patterns prevalent in the Southern US, characterized by high sodium and low nutrient density, may partly explain the higher HTN incidence among Black adults compared to White adults, despite similar rates of dietary counseling [2].

The 2025 AHA/ACC HBP guideline recommends adults with or without HTN to limit dietary sodium intake to < 2300 mg/day, ideally approaching < 1500 mg/day, to prevent or treat elevated BP and HTN [2]. Additionally, potassium-enriched salt substitutes are recommended for individuals whose sodium intake arises primarily from home food preparation, provided there is no contraindication such as CKD or concurrent use of potassium-sparing medications requiring serum potassium monitoring. Evidence supporting this approach is robust. In a large trial in China (*N* = 20,995 adults), use of potassium-based salt substitutes was associated with lower rates of stroke, major CV events, and all-cause mortality compared with regular salt [45]. Furthermore, in a cluster-randomized trial in Peru, replacement of regular salt with a 75% sodium chloride/25% potassium chloride substitute led to modest reductions in BP and a 51% lower risk of developing HTN, supporting the effectiveness of population-wide salt-substitution strategies for HTN prevention [46].

Telehealth strategies have shown promise in improving HTN control. Interventions that integrate home BP monitoring, remote data transmission, lifestyle support, and medication management tend to achieve greater reductions in systolic and diastolic BP and higher rates of HTN control compared with standard clinic-based care. While outcomes in Black and Hispanic populations appear promising, challenges remain - such as disparities in internet access, digital literacy, and integration with EHR - limiting widespread adoption [2]. More research is needed, especially among high-risk and underrepresented groups.

## Pharmacotherapeutic Approaches Across Racial/Ethnic Populations in the 2025 AHA/ACC High Blood Pressure Guideline

**Fig. 1:**
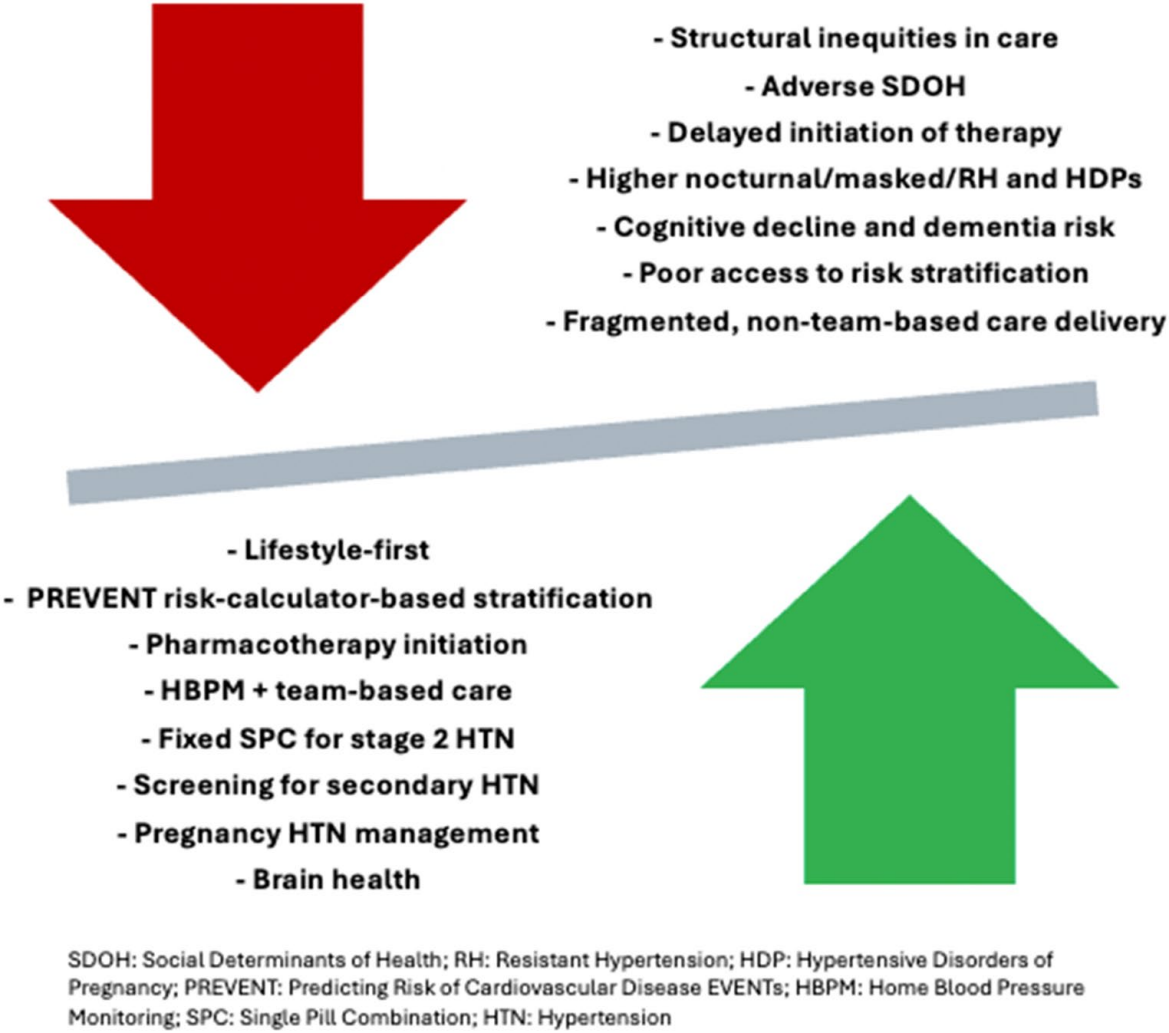
Factors contributing to and improving HTN-related outcomes in racial/ethnic populations

Although there is no specific advice related to drug therapy and race/ethnicity in BP control, the 2025 AHA/ACA HBP guideline provides recommendations that, if applied effectively, should lower BP and risk across all populations, regardless of self-identified race/ethnicity (Fig. 1). Recent clinical trial evidence and evolving guideline recommendations have significantly shaped contemporary approaches to HTN management, highlighting the benefits of intensive BP lowering and the strategic use of combination pharmacotherapy, particularly in high-risk populations. At least five major trials have investigated the impact of intensive BP-lowering in individuals with systolic BP ≥ 130 mmHg and elevated CV risk [47]. Four of the five trials found that lowering SBP below 120–130 mmHg significantly reduced primary CV outcomes, with three also reporting reductions in CV and all-cause mortality [47]. For example, China’s Blood Pressure Control Target in Diabetes (BPROAD) trial (*N* = 12,821) showed that targeting BP control (< 130/80 mmHg) significantly reduced CV events, particularly in patients with T2D [48]. Reflecting this evidence, the 2025 AHA/ACC HBP guideline provides a Class I recommendation for a BP target of < 130/80 mmHg in adults with confirmed HTN and a 10-year CVD risk ≥ 7.5% (as assessed by PREVENT). In adults with stage 1 HTN and presence of CKD or T2D, similar targets may be considered reasonable. Importantly, the guidelines emphasize individualization of treatment goals based on medication tolerance, comorbidities, and life expectancy.

Furthermore, the 2025 AHA/ACC HBP guideline emphasizes the role of single-pill combination (SPC) therapy, especially for patients with stage 2 HTN (≥ 140/90 mmHg) or selected high-risk patients with stage 1 HTN (e.g. NHB adults or ASCVD risk > 7.5%). Initiating treatment with two first-line agents from different classes, ideally in an SPC form, improves BP control and patient adherence [2]. A study evaluating triple SPC containing telmisartan, amlodipine, and indapamide showed superior BP control compared to dual therapy, with improved tolerability and adherence due to reduced pill burden [49]. A study in Nigeria likewise showed promise for this approach in a resource-limited setting [50]. These findings suggest that upfront multi-drug strategies may offer advantages, particularly in under-resourced settings where treatment adherence and access pose challenges. Notably, one recommendation related to race/ethnicity in the 2025 AHA/ACA HBP guideline is that in self-identified Black patients with advanced HF with New York Heart Association class III–IV heart failure with reduced ejection fraction (HFrEF) already on optimal guideline-directed medial therapy, the addition of hydralazine and isosorbide-dinitrate is recommended to improve symptoms and reduce morbidity and mortality [2, 51].

## Pharmacotherapeutic Approaches Across Racial/Ethnic Populations in International Guidelines

Despite the absence of explicit race/ethnicity pharmacotherapy recommendations in the 2025 AHA/ACC HBP guideline, several other international guidelines have reflected some unique considerations for Black adults. The 2020 International Society of Hypertension recommends annual BP screening from age 18, lifestyle interventions, and first-line therapy with a thiazide-like diuretic plus a calcium channel blocker (CCB), or a CCB plus an angiotensin receptor blocker (ARB), with ARBs preferred over angiotensin-converting enzyme (ACE) inhibitors due to the higher risk of angioedema in Black patients [52]. Similarly, the 2019 National Institute for Health and Care Excellence (NICE) advice CCBs as first-line therapy for Black adults without T2D, and ACE inhibitors or ARBs for those with T2D, often in combination due to lower monotherapy efficacy [10]. The 2024 European Society of Cardiology Guidelines highlight low-renin, salt-sensitive HTN and elevated risk of organ damage in Black patients, recommending diuretics or CCBs, alone or with renin–angiotensin system (RAS) blockers, while noting reduced efficacy of RAS blocker monotherapy [53]. The guideline also notes that when using RAS inhibitors in combination therapy, ARBs may be preferred over ACE inhibitors due to greater observed incidence of ACE inhibitor-associated angioedema in Black patients [53]. Across guidelines, attention to NH, salt restriction, and preference for ARBs over ACE inhibitors are emphasized to optimize outcomes and reduce target organ damage.

## Racial and Ethnic Disparities in Hypertension Care

Access disparities remain substantial, with higher rates of uninsured individuals among NHB (9.6%) and Hispanic (16.7%) adults compared to NHW (5.2%) or Asian (6.2%) adults [7]. Black and Hispanic adults had worse access to care than White adults for 50–75% of access measures [7]. Having a usual source of care strongly predicts BP control (48.4% vs. 26.5%) [7]. Even after adjusting for access to care, education, and obesity, disparities in BP control between NHB and NHW adults persisted, suggesting that quality of care factors contribute substantially. In a study among safety-net patients with HTN (*N* = 16,114), Black patients had lower treatment intensification, more missed visits, and lower odd of BP control (OR 0.82), while Asian patients had higher treatment intensification, fewer missed visits, and higher odds of control (OR 1.13). Differences in treatment intensification and visit attendance explained roughly one-third of the observed racial disparities in BP control [54].

Healthcare clinicians and systems are central to reducing HTN disparities through antiracism initiatives, team-based care, and culturally tailored interventions. The AHA recommends multidisciplinary teams, including primary and specialty care physicians, advanced practice nurses, nurse practitioners, nurses, physician assistants, dieticians, pharmacists, case managers, physical therapists, social workers, and community health workers (CHWs) [55]. Evidence shows that team-based care improves BP control, particularly in racial/ethnic populations. Community health workers serve as critical bridges between communities and healthcare systems, and interventions that integrate them into care teams have demonstrated effectiveness in improving BP control, particularly within Black, Hispanic, and Latino populations [55].

Lifestyle modifications remain the cornerstone of BP control across all racial/ethnic groups. The PREVENT tool enables clinicians to deliver individualized CVD prevention, and when paired with SDOH assessment, interventions can be tailored to address barriers such as food insecurity, transportation, and language access [56].

## Conclusion

Racial/ethnic disparities, specifically in the US, in HTN prevalence and management are rooted in long-standing structural inequities. Elevated rates of nocturnal, masked, and RH, as well as HDPs, contribute to disproportionately high CV morbidity and mortality in these groups. Black adults also experience higher rates of cerebrovascular disease and dementia, further compounding CV health disparities driven by HTN.

The 2025 AHA/ACC HBP guideline emphasizes early detection, risk-based tools such as the PREVENT calculator, lifestyle interventions, and multidisciplinary care as essential strategies to address these disparities. Achieving equitable HTN outcomes requires clearer treatment thresholds for diverse populations, inclusive and disaggregated data collection, and targeted research examining the intersection of BP, race/ethnicity, and the SDOH. Health systems, clinicians, and communities must collaborate to implement culturally tailored interventions, antiracism initiatives, and standardized treatment protocols to dismantle systemic barriers and advance CV health equity. Ultimately, health disparities in HTN and CVD reflect structural inequities rather than individual behaviors. Health care organizations can play a pivotal role by addressing inequities at the point of care, reducing institutional bias, and engaging communities, guided by frameworks such as the Institute for Healthcare Improvement’s (IHI) five-component approach to achieving health equity [57]. We must strive to achieve equitable improvements in care for all individuals, irrespective of race/ethnicity, sex/gender, geography, socioeconomic status, or disability/ability. Achieving equity is both a practical necessity and moral imperative [58].

## Key References

- Jones et al., 2015 - Comprehensive and most recent AHA/ACC clinical practice guideline outlining current recommendations for the prevention, detection, evaluation, and management of high blood pressure in adults.
- Muntner et al., 2019 - A foundational AHA scientific statement detailing standardized approaches for accurate blood pressure measurement in clinical and research settings.
- Kario et al., 2024 - Summary and analysis of the WHO 2023 Global Hypertension Report, highlighting the rising global hypertension burden and recommended treatment strategies.
- Khan et al., 2024 - Development and validation of the AHA PREVENT cardiovascular risk equations, offering an updated tool for 10-year and 30-year CVD risk estimation.

## Data Availability

No datasets were generated or analysed during the current study.
